# Tumor Size and Computed Tomography Attenuation of Pulmonary Pure Ground-Glass Nodules Are Useful for Predicting Pathological Invasiveness

**DOI:** 10.1371/journal.pone.0097867

**Published:** 2014-05-20

**Authors:** Takashi Eguchi, Akihiko Yoshizawa, Satoshi Kawakami, Hirotaka Kumeda, Tetsuya Umesaki, Hiroyuki Agatsuma, Takao Sakaizawa, Yoshiaki Tominaga, Masayuki Toishi, Masahiro Hashizume, Takayuki Shiina, Kazuo Yoshida, Shiho Asaka, Mina Matsushita, Tomonobu Koizumi

**Affiliations:** 1 Division of Thoracic Surgery, Department of Surgery, Shinshu University, Matsumoto, Japan; 2 Department of Pathology, Shinshu University, Matsumoto, Japan; 3 Department of Radiology, Shinshu University, Matsumoto, Japan; 4 Comprehensive Cancer Center, Shinshu University, Matsumoto, Japan; Memorial Sloan-Kettering Cancer Center, United States of America

## Abstract

**Objectives:**

Pulmonary ground-glass nodules (GGNs) are occasionally diagnosed as invasive adenocarcinomas. This study aimed to evaluate the clinicopathological features of patients with pulmonary GGNs to identify factors predictive of pathological invasion.

**Methods:**

We retrospectively evaluated 101 pulmonary GGNs resected between July 2006 and November 2013 and pathologically classified them as adenocarcinoma *in situ* (AIS; n = 47), minimally invasive adenocarcinoma (MIA; n = 30), or invasive adenocarcinoma (I-ADC; n = 24). The age, sex, smoking history, tumor size, and computed tomography (CT) attenuation of the 3 groups were compared. Receiver operating characteristic (ROC) curve analyses were performed to identify factors that could predict the presence of pathologically invasive adenocarcinomas.

**Results:**

Tumor size was significantly larger in the MIA and I-ADC groups than in the AIS group. CT attenuation was significantly greater in the I-ADC group than in the AIS and MIA groups. In ROC curve analyses, the sensitivity and specificity of tumor size (cutoff, 11 mm) were 95.8% and 46.8%, respectively, and those for CT attenuation (cutoff, −680 HU) were 95.8% and 35.1%, respectively; the areas under the curve (AUC) were 0.75 and 0.77, respectively. A combination of tumor size and CT attenuation (cutoffs of 11 mm and −680 HU for tumor size and CT attenuation, respectively) yielded in a sensitivity and specificity of 91.7% and 71.4%, respectively, with an AUC of 0.82.

**Conclusions:**

Tumor **s**ize and CT attenuation were predictive factors of pathological invasiveness for pulmonary GGNs. Use of a combination of tumor size and CT attenuation facilitated more accurate prediction of invasive adenocarcinoma than the use of these factors independently.

## Introduction

We have previously evaluated the usefulness of computed tomography (CT) as a screening tool for lung cancer [1], [2]. With the increased use of CT screening, cases of lung cancer appearing as pure ground-glass nodules (GGNs), which are radiologically nonsolid nodules, are being detected with increasing frequency [3]. Neoplastic cells in pure GGNs are usually distributed along pre-existing alveolar structures in a lepidic growth pattern without interstitial invasion [4], and because of this, limited resection is sometimes indicated in patients with pulmonary pure GGNs. However, a subset of pulmonary pure GGNs are associated with pathological invasion, and, in general, it is difficult to distinguish between pure GGNs with invasion and those without invasion by CT examination. The relationship between pathological invasiveness and radiological findings of pulmonary pure GGNs has not yet been fully elucidated, and hence, the objective of this study was to evaluate the demographic and clinicopathological features of patients with pulmonary pure GGNs in order to identify factors predictive of pathological invasion.

## Patients and Methods

This retrospective study was approved by the institutional review board of Shinshu University Hospital, Matsumoto, Japan, and was conducted in accordance with the principles outlined in the Declaration of Helsinki.

Between July 2006 and November 2013, 775 patients underwent lung resection for primary lung cancer at Shinshu University Hospital. Among these patients, 101 tumors in 98 patients appeared as pure GGNs on the last CT examination performed before surgery, and we retrospectively investigated the clinicopathological characteristics of these 101 tumors. During this period, we used 2 types of CT scanners for the diagnosis of GGNs: Light Speed Ultra (GE Healthcare, Freiburg, Germany) CT scanner from July 2006 to December 2007; and the Light Speed VCT Vision (GE Healthcare) CT scanner from December 2007 onwards. Written informed consent was not given by participants for their clinical records to be used in this study. Patient records/information was anonymized and de-identified prior to analysis.

### Radiological Definition

All CT examinations were performed at our institute, and full resolution scans of 1.25-mm-thick sections were obtained without the use of contrast media. All tumors were viewed in both the lung window setting (window level, −550 Hounsfield units [HU]; window width, 1500 HU) and mediastinal window setting (window level, 30 HU; window width, 400 HU). Two experienced radiologists (SK and MM), who were blinded to the patients’ clinical information, independently interpreted all of the scans.

Pure GGNs were defined as focal nodular areas of increased lung attenuation, through which normal parenchymal structures, including airways and vessels, could be visualized [5]. Nodules that included both ground-glass and solid components were defined to be ‘part-solid GGNs’ [6], and were excluded from the study. Solid components were evaluated using the mediastinal window setting [6]. The radiological tumor size was defined as the maximum lesion diameter in the lung window setting. The mean CT attenuation was measured using the region-of-interest cursors, which traced the edge of the tumor on the slices containing the region of the lesion with the maximum diameter [7].

### Histological Examination

All tumors were histologically evaluated by two experienced pathologists (AY and SA), who were blinded to the patients’ clinical information. All histological evaluations were performed by examining hematoxylin and eosin stained slides which were prepared using formalin-fixed paraffin-embedded tissues. Adenocarcinoma lesions were classified according to the new lung adenocarcinoma classification proposed by the International Association for the Study of Lung Cancer, American Thoracic Society, and European Respiratory Society (IASLC/ATS/ERS) [8].

### Surgical Criteria

In our institute, surgical resection for pure GGNs was indicated in cases of tumors ≥10 mm in size, tumors enlarged during the follow-up period, and other tumors with surgical indications present in the ipsilateral lung. The surgical procedure for pure GGNs was determined according to the tumor size as follows: <10 mm, partial resection; 10 mm to <20 mm, segmentectomy or lobectomy; and ≥20 mm, lobectomy. However, radiological follow-up or limited surgery were occasionally selected by the attending physicians on considering factors such as patient age, lung function, performance status, and pre-existing diseases.

### Statistical Analysis

The tumors were divided into 3 groups according to pathological classifications: (1) adenocarcinoma *in situ* (AIS), (2) minimally invasive adenocarcinoma (MIA), and (3) invasive adenocarcinoma (I-ADC). Patient characteristics (age, sex, smoking history, and preoperative plasma carcinoembryonic antigen [CEA] levels), tumor size, and mean CT attenuation values were compared between the 3 groups. Continuous variables were compared using the analysis of variance with Scheffe’s post-hoc test, and categorical data were compared using Chi-square tests. Pearson’s correlation analyses were used to verify whether the tumor size and mean CT attenuation values could be useful for predicting the pathological invasion diameter, and receiver operating characteristic (ROC) curve analyses were used to confirm the predictive value of factors identified as predictive of pathologically invasive adenocarcinomas. All statistical analyses were performed using PASW Statistics 18.0 (IBM corp., Armonk, NY). All data are reported as mean ± standard deviation, and statistical significance was set at *P*<0.05.

## Results

### Patient Characteristics and Radiological Tumor Properties

The age, sex, smoking history, serum CEA levels, tumor size, and mean CT attenuation for all patients and for each group are summarized in Table 1.

**Table 1:** Patient characteristics and tumor properties.

	Total (n = 103)	AIS (n = 47)	MIA (n = 30)	I- ADC (n = 24)	p value
Age (years)	64.3±9.7	63±10.8	64.3±9.2	66.3±8	0.3953
Sex (male/female)	39/62	19/28	14/16	6/18	0.5983
Smoking history (with/without)	31/70	15/32	13/17	3/21	0.1976
CEA level (ng/ml)	2.1±1.7	1.8±0.9	2.6±2.6	2.1±1.1	0.1408
Tumor size (mm)	13.1±5.5	11±3.6	14.7±7.6#	15.5±3.6#	0.0005
CT attenuation (HU)	−621±86.3	−649.8±88.4	−625.8±88.4	−560.2±69.3*	<0.0001

Abbreviations: AIS, adenocarcinoma in situ; MIA, minimally invasive adenocarcinoma; I-ADC, invasive adenocarcinoma; CEA, carcinoembryonic antigen; CT, computed tomography; HU, Hounsfield unit.

# significantly higher than tumor size in the AIS group.

*significantly higher than CT attenuation in the AIS and MIA groups.

### Surgical Procedures

The surgical procedures performed for the 101 tumors in 98 patients were as follows: lobectomy, 34 tumors in 32 patients; segmentectomy, 28 tumors in 28 patients; and partial resection, 39 tumors in 39 patients. In 2 patients who underwent lobectomy, 2 tumors were present in the same lobe. In 1 patient with two tumors in different lobes in the ipsilateral lung, lobectomy and partial resection were performed.

### Pathological Findings and Outcomes

Pathologically, all lesions were diagnosed as pulmonary adenocarcinoma. The IASLC/ATS/ERS classification of these adenocarcinomas was as follows: AIS, 47 lesions; MIA, 30 lesions; and I-ADC, 24 lesions (lepidic predominant, 10 lesions; papillary predominant, 8 lesions; and acinar predominant, 6 lesions). The mean pathological maximum diameter was 11.0±5.4 mm. The mean invasion diameter was 2.9±3.7 mm. All patients in our study were classified as having pathological stage T1aN0M0 lung cancer. No vascular invasion, lymph node metastasis, or intrapulmonary metastasis was detected.

**Figure 1:**
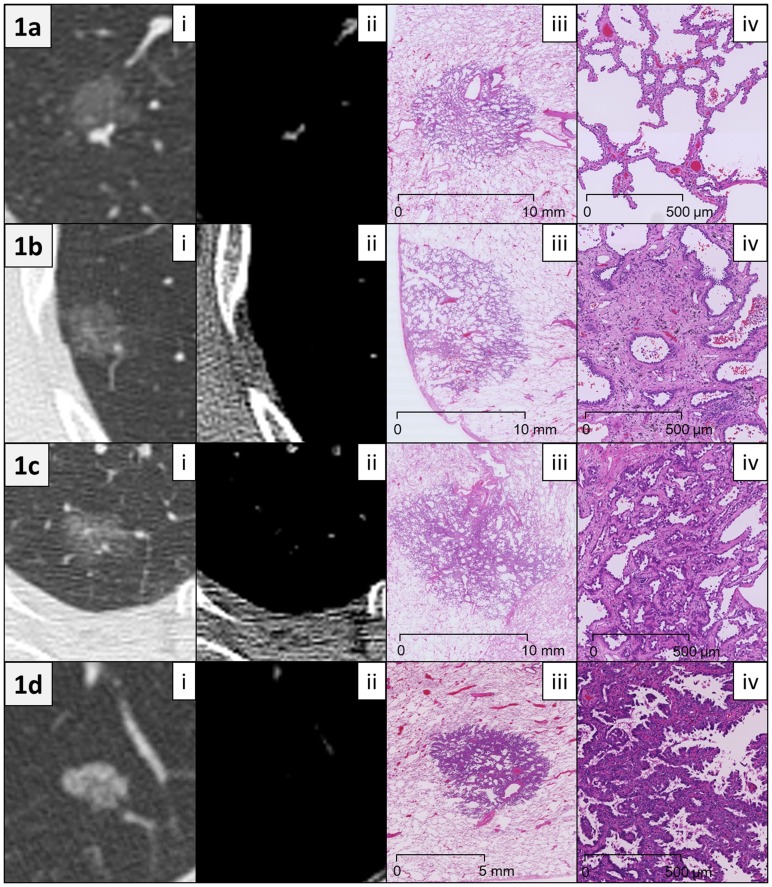
Representative radiological and histological images. (a) A 64-year-old female patient with adenocarcinoma *in situ*. (i) Computed tomography (CT) scan (lung window setting) showed a pure ground-glass nodule (GGN), 11.9 mm in size. The mean CT attenuation of the tumor was −716 Hounsfield units (HU). (ii) Mediastinal window setting CT showed no tumor components except for vessels. (iii) Low magnification image (hematoxylin and eosin (HE) staining) showed a circumscribed tumor growing purely with a lepidic pattern without foci of invasion. A slight thickening of the alveolar walls in the tumor area was observed. (iv) Middle magnification image of the tumor (HE staining) revealed that tumor cells appeared to replace normal pneumocytes on alveolar walls. (b) A 63-year-old female patient with minimally invasive adenocarcinoma. (i) Lung window CT image showed a pure GGN, 14.2 mm in size. The mean CT attenuation was −691 HU. (ii) Mediastinal window CT showed no tumor components. (iii) Low magnification image of the tumor (HE staining) revealed a subpleural tumor consisting predominantly of lepidic growth with a small (<5 mm) focus of invasion. (iv) Middle magnification image of the invasive area of the tumor (HE staining) revealed acinar-type growth pattern. (c) A 74-year-old female patient with lepidic-predominant invasive adenocarcinoma. (i) Lung window CT showed a pure GGN, 19.7 mm in size. The mean CT attenuation was −618 HU. (ii) Mediastinal window CT showed no tumor components except for vessels. (iii) Low magnification image (HE staining) revealed a tumor consisting mostly of lepidic growth with a smaller area (8 mm) of acinar invasion. (iv) Middle magnification image of the invasion area of the tumor (HE staining) revealed acinar gland proliferation in the fibrous stroma. (d) A 76-year-old male patient with papillary-predominant invasive adenocarcinoma. (i) Lung window CT image showed a pure GGN, 10.7 mm in size. The mean CT attenuation was −509 HU. (ii) Mediastinal window CT showed no tumor components. (iii) Low magnification image of the tumor (HE staining) revealed that the tumor predominantly consisted of papillary proliferation. (iv) Middle magnification image of the tumor (HE staining) revealed cuboidal tumor cells growing along fibrovascular cores in a papillary configuration.

The median follow-up duration (from the date of surgery to the last follow-up) was 21.2 months (range 1–51.2 months). Recurrence was not observed in any patient, and 1 patient in the MIA group died 12 months after surgery due to an unrelated cause. Figure 1 shows representative radiological and histological images from patients in each of the groups.

### Comparison of Demographic and Clinicopathological Characteristics

The patient characteristics and tumor properties for all patients and for patients in each group are shown in Table 1. When comparing the 3 groups, the tumor size and mean CT attenuation significantly differed between the groups (p = 0.0005 and p<0.0001, respectively). Tumor size was significantly larger in the MIA and I-ADC groups than in the AIS group (p = 0.0100 and 0.0025, respectively). Mean CT attenuation was significantly higher in the I-ADC group than in the AIS and MIA groups (p = 0.0001 and 0.0129, respectively).

### Pearson’s Correlation Analyses between the Pathological Invasion Diameter and the Tumor Size/CT Attenuation

The correlation coefficients for the pathological invasion diameter with tumor size and CT attenuation were 0.3740 and 0.4072 (p = 0.0001 and p<0.0001, respectively).

### ROC Curve Analyses of Tumor Size and CT Attenuation for Predicting Invasive Adenocarcinoma

**Figure 2:**
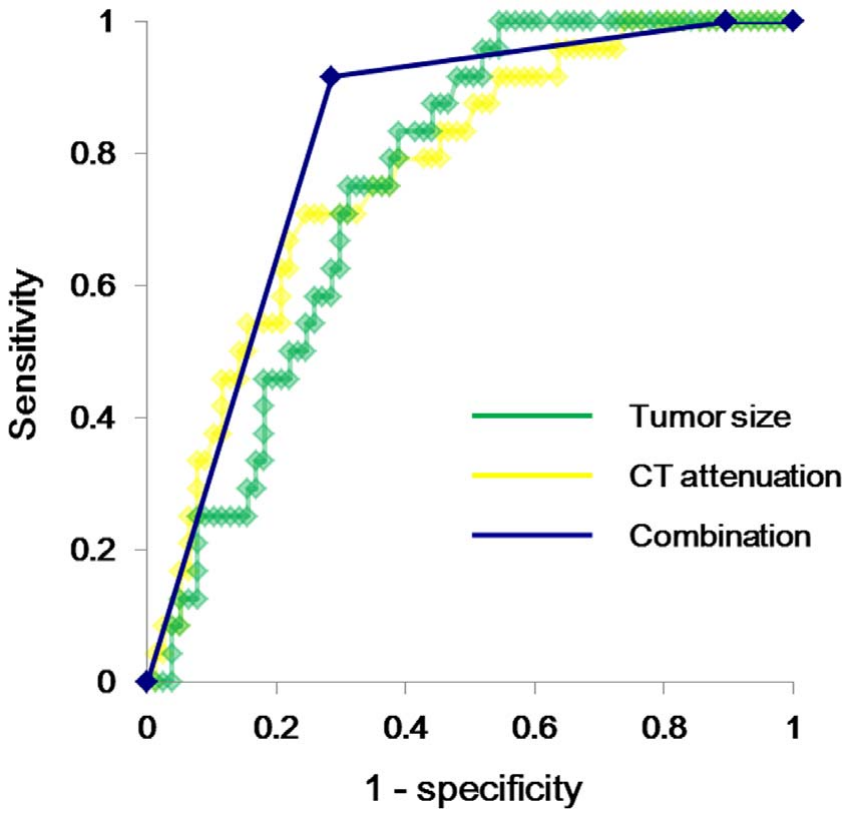
Receiver operating characteristic curve analysis for invasive adenocarcinoma prediction: tumor size and computed tomography attenuation. The sensitivity and specificity of tumor size for predicting invasive adenocarcinoma were 95.8% and 46.8%, respectively, at a cutoff value of 11.0 mm, with an area under the curve (AUC) of 0.75 (green curve). The sensitivity and specificity of the mean computed tomography (CT) attenuation were 95.8% and 35.1%, respectively, at a cutoff value of –680 HU, with an AUC of 0.77 (yellow curve). The sensitivity and specificity of the combined variable (tumor size and mean CT attenuation) were 91.7% and 71.4%, respectively, at cutoff values of 11 mm and −680 HU, with an AUC of 0.82 (blue curve).

With regard to tumor size as a predictor of invasive adenocarcinoma, the highest odds ratio (20.2) was obtained at a cutoff value of 11.0 mm, and the sensitivity and specificity were 95.8% and 46.8%, respectively, with an area under the curve (AUC) of 0.75 (Figure 2). With regard to CT attenuation, the highest odds ratio (12.4) was obtained at a cutoff value of –680 HU, and the sensitivity and specificity were 95.8% and 35.1%, respectively, with an AUC of 0.77 (Figure 2). A combined variable was constructed using these 2 cutoff values:

- Group A: Neither tumor size nor mean CT attenuation indicated invasive adenocarcinoma (i.e., tumor size ≤11 mm and mean CT attenuation ≤–680 HU)
- Group B: Either tumor size or mean CT attenuation indicated invasive adenocarcinoma (i.e., tumor size >11 mm or mean CT attenuation >–680 HU)
- Group C: Both tumor size and mean CT attenuation indicated invasive adenocarcinoma (i.e., tumor size >11 mm and mean CT attenuation >–680 HU)

The sensitivity and specificity of the combined variable for predicting invasive adenocarcinoma at the cutoff values were 100% and 10.4% for group B and 91.7% and 71.4% for group C, respectively, with an AUC of 0.82 (Figure 2).

## Discussion

In this present study, we showed that approximately half of all resected pulmonary pure GGNs displayed a pathological invasive area. Moreover, approximately a quarter of the resected pure GGNs were diagnosed as invasive adenocarcinomas, and we found that tumor size and mean CT attenuation were useful in predicting pathological invasiveness.

We have previously reported a strong negative association between CT attenuation and retained air space in tumors in a study of small peripheral lung adenocarcinomas detected by CT screening [7]. We moreover found that the retained air space was larger in non-invasive adenocarcinomas than in invasive adenocarcinomas and that this was largely due to an increased tumor tissue component and thickening of the alveolar septa in invasive adenocarcinomas, resulting in reduced air space. These findings suggested that the high CT attenuation of pure GGNs reflects the large number of tumor cells that grow along alveolar septa, and, in terms of a stepwise progression of adenocarcinoma, indicates that the lesion is progressing to invasive adenocarcinoma [4].

In general, an invasive area is thought to be nonaerated and is theoretically thought to appear as a solid component on high-resolution CT. In the present study, however, a subset of pure GGNs were pathologically diagnosed as invasive adenocarcinoma. This discrepancy between radiological and pathological findings could be explained by a partial volume effect, which means that, when the slice thickness is relatively high (2–2.5 mm), detection of small nonaerated components may be difficult because of inadequate spatial resolution [9], [10], [11]. Although, all CT images were acquired by using high resolution CT at a 1.25-mm slice thickness in the present study, a much higher resolution may be needed to detect small nonaerated invasion. Meanwhile, in some invasive adenocarcinomas, sparsely proliferated areas are histologically recognized in papillary or acinar lesions, and these sparsely invasive lesions could be aerated and thus depicted as nonsolid portions on CT images.

Recently, limited resection for early-stage lung cancers has been advocated for preserving lung function [12]. However, there are currently no established criteria for the indication of limited resection. Suzuki, et al. demonstrated the prognostic importance of the extent of central fibrosis. In their study, the 5-year survival rate was reported to be 100% if the region of central fibrosis was ≤5 mm, 72% if the region was between 5 and 15 mm, and 40% if the region was >15 mm [13]. In the new adenocarcinoma classification proposed by IASLC/ATS/ERS in 2011, a new concept of minimally invasive adenocarcinoma for lepidic predominant tumors with ≤5-mm invasion was recommended in order to distinguish these tumors from invasive adenocarcinomas, in which invasion is >5 mm [8]. Yoshizawa, et al. reported in their analysis of 514 stage I adenocarcinoma cases that AIS and MIA were associated with a 100% 5-year disease-free survival rate after complete resection [14]. These findings suggest that limited resection might be indicated for adenocarcinoma cases classified as AIS/MIA. For feasibility in clinical practice, accurate preoperative differentiation of AIS/MIA from invasive adenocarcinoma is important.

In the present study, upon ROC curve analyses for predicting invasive adenocarcinoma, tumor size and mean CT attenuation yielded AUC values of 0.75 and 0.77, respectively. AUC values between 0.7 and 0.9 are thought to indicate a moderately accurate test [15], suggesting that these two parameters may represent useful predictors of invasive adenocarcinoma in cases of pulmonary pure GGNs. Moreover, ROC curve analysis of the combination of the two factors resulted in an AUC of 0.82, suggesting that the use of this combination may facilitate more accurate prediction of invasive adenocarcinoma than the use of these factors individually. However, the usefulness of tumor size and CT attenuation as predictive factors for the invasiveness of pure GGNs should be confirmed in further large-scale studies, and additional accurate tests should be established in order to facilitate preoperative decision making regarding surgical procedures for pulmonary pure GGNs.

This present study has a number of limitations. First, the small number of patients analyzed and the retrospective nature of the analysis may have affected our results. A second limitation of our study was that the mean CT attenuation value of pure GGNs was evaluated only in the slice containing the part of the lesion with its maximum diameter, and thus, this value could potentially be quite different from that of the entire tumor.

## Conclusions

A subset of pulmonary pure GGNs have histological invasive areas that cannot be recognized as solid components on CT, and, in this study, we found that tumor **s**ize and CT attenuation in cases of pure GGNs could successfully predict pathological invasiveness. The combination of tumor size and CT attenuation might enable a more accurate prediction of invasive adenocarcinoma than the two factors individually, and further, large-scale studies should be conducted to confirm these results.
